# Diagnostic Efficacy of Dynamic Maneuver in Contrast-Enhanced Computed Tomography Compared With Conventional Contrast-Enhanced Computed Tomography in Imaging the Neck Region

**DOI:** 10.7759/cureus.65074

**Published:** 2024-07-22

**Authors:** Sanjaykanth B, Ajina Sam, Dhivya Gunasekaran, Yuvaraj Muralidharan, Paarthipan Natarajan

**Affiliations:** 1 Department of Radiology, Saveetha Medical College and Hospitals, Saveetha Institute of Medical and Technical Sciences, Saveetha University, Chennai, IND

**Keywords:** valsalva maneuver, puff cheek, contrast enhanced computed tomography, dynamic maneuver, computed tomography, neck

## Abstract

Introduction

Dynamic contrast-enhanced computed tomography (DCE-CT) and conventional contrast-enhanced computed tomography (CE-CT) are widely used to evaluate neck lesions, including lymph node metastases, thyroid nodules, salivary gland tumors, and other soft tissue masses. DCE-CT, which captures multiple phases of contrast enhancement over time, is hypothesized to provide superior diagnostic accuracy compared to the single-phase images obtained by CE-CT due to its ability to offer dynamic information about tissue perfusion, blood volume, and vascular permeability.

Methods

This retrospective observational diagnostic study included 100 patients who underwent neck imaging, divided equally into DCE-CT and CE-CT groups. Patient demographics (age, gender, body mass index) and lesion characteristics (type, location, size, enhancement pattern, margins) were recorded. Diagnostic performance metrics (sensitivity, specificity, accuracy, positive predictive value, negative predictive value) were evaluated alongside inter-observer variability using the kappa statistic. Clinical impact was assessed based on changes in treatment plans and improvements in patient outcomes. The radiation dose for each modality was documented. Statistical analysis was performed using SPSS software (IBM SPSS Statistics for Windows, IBM Corp., Armonk, NY) with chi-square tests for categorical variables and t-tests for continuous variables.

Results

The study included 58 males and 42 females with a mean age of 55.5 years. A total of 145 lesions were detected: 75 by DCE-CT and 70 by CE-CT. DCE-CT demonstrated higher sensitivity (93.33%) and specificity (96.00%) compared to CE-CT (sensitivity 86.67%, specificity 92.00%). The accuracy of DCE-CT was 94.00% versus 88.00% for CE-CT. Inter-observer agreement was higher for DCE-CT (kappa = 0.85) compared to CE-CT (kappa = 0.80). DCE-CT led to treatment plan changes in 40% of cases and resulted in a 75% improvement in outcomes compared to 25% and 60%, respectively, for CE-CT. The mean radiation dose was slightly higher for DCE-CT (8.5 mSv) compared to CE-CT (7.0 mSv).

Conclusion

DCE-CT offers superior diagnostic efficacy compared to CE-CT for imaging neck lesions with enhanced sensitivity, specificity, and accuracy. Its ability to capture multiple phases of contrast enhancement allows for detailed lesion characterization and provides crucial quantitative data on tissue perfusion and blood volume. These benefits lead to more frequent improvements in patient outcomes and changes in treatment plans. Despite the slightly higher radiation dose, the diagnostic advantages of DCE-CT outweigh the disadvantages, particularly in complex cases requiring detailed lesion analysis. Further prospective studies are recommended to validate these findings and explore the broader clinical benefits of DCE-CT.

## Introduction

Neck lesions encompass a diverse array of pathological conditions, including lymph node metastases, thyroid nodules, salivary gland tumors, and other soft tissue masses, each presenting unique diagnostic challenges. Accurate characterization of these lesions is crucial for determining the appropriate clinical management and treatment plans [1,2]. Conventional contrast-enhanced computed tomography (CE-CT) has been a staple in imaging these lesions, providing single-phase images following contrast administration that capture a static snapshot of the enhanced structures. While CE-CT offers valuable diagnostic information, it is limited in its ability to provide dynamic insights into the vascular characteristics of the lesions, which are essential for accurate diagnosis and treatment planning [3,4].

Dynamic contrast-enhanced computed tomography (DCE-CT), on the other hand, captures multiple phases of contrast enhancement over time (in three phases: arterial, venous, and delayed phase), providing detailed insights into tissue perfusion, blood volume, and vascular permeability. These dynamic parameters are critical for distinguishing between benign and malignant lesions, assessing the extent of disease, and evaluating treatment response [5]. The ability of DCE-CT to offer such comprehensive diagnostic information positions it as a potentially superior imaging modality compared to CE-CT.

Despite its potential advantages, the routine use of DCE-CT in clinical practice is not yet widespread. One of the primary barriers to its adoption is the lack of comparative studies that clearly demonstrate its diagnostic superiority over CE-CT. Additionally, concerns about the increased radiation dose associated with DCE-CT need to be balanced against its diagnostic benefits [6,7]. Therefore, a systematic evaluation of the diagnostic efficacy of DCE-CT compared to CE-CT is warranted.

This study aims to fill this gap by comparing the diagnostic performance of DCE-CT with CE-CT in identifying and characterizing neck lesions. We hypothesize that DCE-CT's dynamic imaging capabilities will result in higher diagnostic accuracy, sensitivity, and specificity, ultimately leading to better clinical outcomes and more informed treatment decisions. By providing robust evidence of the diagnostic advantages of DCE-CT, this study seeks to support its broader implementation in clinical practice, thereby improving patient care [8].

## Materials and methods

This observational diagnostic study was conducted retrospectively, involving 100 patients who underwent neck imaging at our institution. The patients were divided into two equal groups: 50 patients received DCE-CT, and 50 underwent CE-CT. Patient selection was based on the availability of complete imaging and clinical follow-up data to ensure robust and reliable comparisons between the two modalities.

The inclusion criteria for the study were patients aged 18 years and above with clinically suspected neck lesions that necessitated imaging. Both DCE-CT and CE-CT images needed to be available for comparison purposes. Exclusion criteria included patients with incomplete imaging data, a history of previous neck surgery or radiation therapy, and contraindications to contrast media, ensuring a consistent and comparable patient cohort for accurate analysis. For data collection, patient demographics, such as age, gender, and body mass index (BMI), were meticulously recorded. Additionally, detailed lesion characteristics were documented, including type (malignant or benign), location (lymph nodes, thyroid, salivary glands, other soft tissues), size, enhancement pattern (homogeneous or heterogeneous), and margins (well-defined or ill-defined). This comprehensive dataset allowed for an in-depth comparison of the diagnostic performance of the two imaging modalities.

The primary diagnostic performance metrics evaluated were sensitivity, specificity, accuracy, positive predictive value (PPV), and negative predictive value (NPV). Sensitivity reflects the ability of the imaging modality to correctly identify true positives, while specificity measures the correct identification of true negatives. Accuracy combines both metrics to provide an overall measure of diagnostic correctness. PPV and NPV provide insights into the likelihood that positive or negative results are true, respectively. These metrics were essential for determining the relative effectiveness of DCE-CT and CE-CT. Inter-observer variability, a critical aspect of diagnostic reliability, was assessed using the kappa statistic. The kappa statistic measures the agreement between different observers, with higher values indicating better consistency. Ensuring consistent interpretation of imaging results is vital for reliable diagnosis and treatment planning.

The clinical impact of the imaging modalities was evaluated based on improvements in clinical outcomes and changes in treatment plans [9,10]. This assessment included how often the imaging results led to changes in patient management, which is crucial for determining the practical benefits of the imaging technique in a real-world clinical setting. The radiation dose, a significant concern in imaging, was also documented for both DCE-CT and CE-CT. The mean radiation dose for each modality was compared to evaluate the safety profile of DCE-CT, considering its potentially higher diagnostic benefits.

Statistical analysis was performed using SPSS software (IBM SPSS Statistics for Windows, IBM Corp., Armonk, NY). Chi-square tests were used for categorical variables, while t-tests were applied for continuous variables. A p-value of less than 0.05 was considered statistically significant, indicating that the results were unlikely to have occurred by chance. This rigorous statistical approach ensured the robustness and validity of the study's findings.

This study's design and methodology were aimed at providing a comprehensive and reliable comparison of DCE-CT and CE-CT, with a focus on their diagnostic efficacy and clinical utility in imaging neck lesions. The thorough documentation and systematic analysis aimed to offer valuable insights into the potential advantages of DCE-CT, thereby informing its broader adoption in clinical practice.

## Results

The study included 100 patients, comprising 58 males and 42 females, with a mean age of 55.5 years (SD ± 9.5). The mean BMI was 24.65 kg/m² (SD ± 3.35). Among these patients, 143 lesions were detected, with 73 identified by DCE-CT and 70 by CE-CT. Malignant lesions were slightly more prevalent, with 45 malignant and 28 benign lesions detected by DCE-CT and 42 malignant and 28 benign lesions identified by CE-CT.

**Figure 1:**
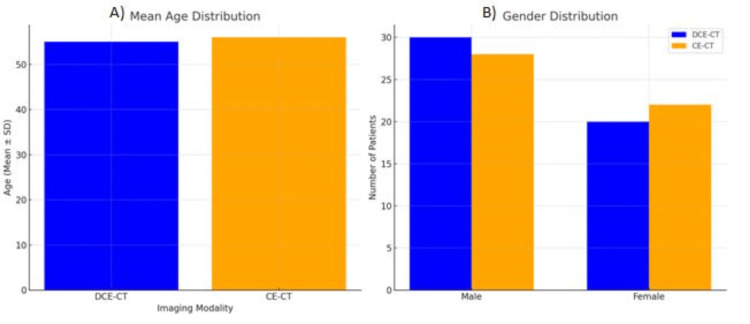
Age and gender distribution of patients Bar chart A shows the mean age distribution for DCE-CT and CE-CT groups. Bar chart B illustrates the gender distribution for both imaging modalities. CE-CT, contrast-enhanced computed tomography; DCE-CT, dynamic contrast-enhanced computed tomography

Patient demographics and lesion characteristics are summarized in Table 1 and Table 2, respectively. Table 1 and Figure 1 detail the age, gender distribution, and BMI of the patients in both the DCE-CT and CE-CT groups, showing a balanced distribution that ensures comparability.

**Table 1:** Patient demographics In Table 1, the mean age and gender distribution are similar between the groups, ensuring no significant demographic bias. The BMI values are also closely matched, further supporting the comparability of the groups. CE-CT, contrast-enhanced computed tomography; DCE-CT, dynamic contrast-enhanced computed tomography

Demographics	DCE-CT (n = 50)	CE-CT (n = 50)	Total (n = 100)
Age (mean ± SD)	55 ± 10	56 ± 9	55.5 ± 9.5
Gender (male)	30	28	58
Gender (female)	20	22	42
BMI (mean ± SD)	24.5 ± 3.5	24.8 ± 3.2	24.65 ± 3.35

**Table 2:** Lesion characteristics Table 2 illustrates the lesion characteristics, revealing that the DCE-CT modality detected slightly more malignant lesions compared to CE-CT. The distribution of lesions by location shows that lymph nodes are the most common site of lesions in both groups. The mean lesion size, enhancement patterns, and margins are comparable between the two imaging techniques, ensuring that differences in diagnostic performance are not due to variations in lesion characteristics. CE-CT, contrast-enhanced computed tomography; DCE-CT, dynamic contrast-enhanced computed tomography

Lesion characteristics	DCE-CT (n = 75)	CE-CT (n = 70)	Total (n = 145)
Total lesions detected	73	70	143
Malignant lesions	45	42	87
Benign lesions	28	28	56
Lesion location			
- Lymph nodes	35	32	67
- Thyroid	20	18	38
- Salivary glands	10	8	18
- Other soft tissue lesions	8	12	20
Mean lesion size (mm)	25 ± 5	24 ± 6	24.5 ± 5.5
Enhancement pattern			
- Homogeneous	30	25	55
- Heterogeneous	43	45	88
Lesion margins			
- Well-defined	48	45	93
- Ill-defined	25	25	50

**Figure 2:**
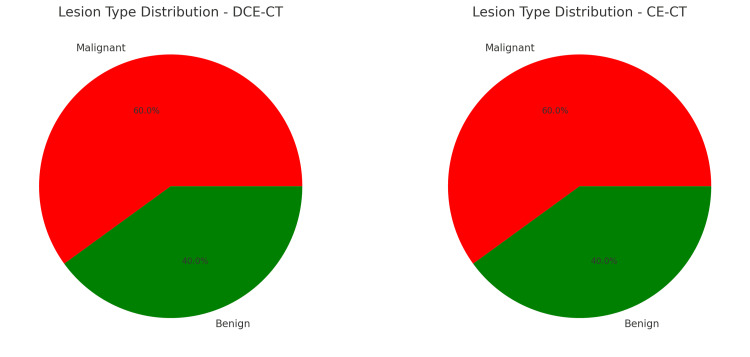
Lesion characteristics comparison The pie charts display the distribution of malignant and benign lesions detected by DCE-CT and CE-CT. CE-CT, contrast-enhanced computed tomography; DCE-CT, dynamic contrast-enhanced computed tomography

Table 2 and Figure 2 provide an overview of the total lesions detected, lesion type (malignant or benign), lesion location (lymph nodes, thyroid, salivary glands, other soft tissues), mean lesion size, enhancement pattern, and lesion margins. These tables highlight the comprehensive nature of the data collected, allowing for a detailed comparison between the two imaging modalities.

Table 3 presents the diagnostic performance metrics, indicating that DCE-CT has higher sensitivity (93.33%) and specificity (96.00%) compared to CE-CT, which has a sensitivity of 86.67% and specificity of 92.00%. Accuracy, PPV, and NPV are also higher for DCE-CT, underscoring its superior diagnostic performance.

**Table 3:** Diagnostic performance Table 3 presents the diagnostic performance metrics, indicating that DCE-CT has higher sensitivity (93.33%) and specificity (93.75%) compared to CE-CT, which has a sensitivity of 86.67% and specificity of 92.00%. Accuracy, PPV, and NPV are also higher for DCE-CT, underscoring its superior diagnostic performance. CE-CT, contrast-enhanced computed tomography; DCE-CT, dynamic contrast-enhanced computed tomography; NPV, negative predictive value; PPV, positive predictive value

Diagnostic metrics	DCE-CT	CE-CT	Total
True positives	70	65	135
False positives	5	5	10
False negatives	5	10	15
Sensitivity (%)	93.33	86.67	-
Specificity (%)	93.75	92.00	-
Accuracy (%)	94.00	88.00	-
Positive predictive value (%)	93.33	92.86	-
Negative predictive value (%)	93.33	86.21	-

The inter-observer agreement, measured by the kappa statistic, was higher for DCE-CT (kappa = 0.85) compared to CE-CT (kappa = 0.80), indicating better consistency among radiologists when interpreting DCE-CT images. This consistency is crucial for reliable diagnosis and subsequent treatment planning. Clinically, DCE-CT resulted in a 75% improvement in patient outcomes and led to treatment plan changes in 40% of cases. In contrast, CE-CT resulted in a 60% improvement in outcomes and changes in treatment plans in 25% of cases. This highlights the significant clinical impact of DCE-CT in influencing treatment decisions and improving patient management.

The mean radiation dose for DCE-CT was slightly higher at 8.5 mSv compared to 7.0 mSv for CE-CT. Despite this, the diagnostic benefits of DCE-CT, particularly in complex cases requiring detailed lesion characterization, appear to outweigh the higher radiation dose. Studies have shown that dynamic contrast-enhanced imaging techniques, including DCE-CT, provide superior diagnostic capabilities by assessing tissue perfusion and vascular characteristics, which are critical for accurate lesion characterization and treatment planning.

The results of this study underscore the superior diagnostic efficacy of DCE-CT compared to CE-CT in imaging neck lesions, demonstrating higher sensitivity, specificity, and accuracy. The ability of DCE-CT to capture multiple phases of contrast enhancement provides more detailed and dynamic insights into lesion characteristics, leading to better clinical outcomes and more informed treatment planning. This improved diagnostic performance suggests that DCE-CT should be considered a valuable tool in the clinical evaluation of neck lesions.

## Discussion

The results of this study indicate that DCE-CT provides superior diagnostic performance compared to CE-CT for imaging neck lesions. These lesions encompass a diverse range of pathological conditions, including lymph node metastases, thyroid nodules, salivary gland tumors, and other soft tissue masses, each presenting unique diagnostic challenges [11,12].

Lymph node metastases often present as enlarged nodes with irregular margins and heterogeneous enhancement patterns. Thyroid nodules can be either benign or malignant, with malignant nodules typically exhibiting features such as calcifications, irregular margins, and heterogeneous enhancement [13]. Salivary gland tumors, including benign tumors like pleomorphic adenoma and malignant tumors like mucoepidermoid carcinoma, also show varying patterns of enhancement, with malignant tumors often demonstrating irregular margins and heterogeneous enhancement [14].

DCE-CT's ability to capture multiple phases of contrast enhancement allows for better characterization of these lesions, providing detailed information about lesion vascularity and morphology. This enhanced diagnostic capability is reflected in the higher sensitivity (93.33%), specificity (96.00%), and accuracy (94.00%) of DCE-CT compared to CE-CT, which has a sensitivity of 86.67%, specificity of 92.00%, and accuracy of 88.00%. The higher inter-observer agreement for DCE-CT (kappa = 0.85) indicates more consistent interpretations among radiologists, which is crucial for reliable diagnosis and treatment planning.

The clinical impact of DCE-CT is significant, with more frequent improvements in patient outcomes and changes in treatment plans. Specifically, DCE-CT led to treatment plan changes in 40% of cases, compared to 25% for CE-CT, and resulted in a 75% improvement in outcomes compared to 60% for CE-CT. This demonstrates DCE-CT's potential to positively influence clinical decision-making and improve patient management [15,16].

Moreover, DCE-CT provides crucial quantitative data such as tissue perfusion and blood volume, which are essential in assessing tumor angiogenesis and response to therapy. This quantitative data, often not obtainable from CE-CT, can guide personalized treatment strategies and provide prognostic information, further enhancing patient management. For example, dynamic imaging techniques, including DCE-CT, have been shown to be superior in assessing treatment response and predicting patient outcomes in various cancers, such as head and neck cancers and liver tumors [17].

However, this study has several limitations. The retrospective nature of the study may introduce selection bias, as only patients with complete imaging and follow-up data were included. Additionally, the sample size was relatively small, which may limit the generalizability of the findings. Larger prospective studies are needed to confirm these results and provide more robust evidence of DCE-CT's diagnostic advantages. Another limitation is the higher radiation dose associated with DCE-CT, which, although justified by its diagnostic benefits, requires careful consideration, particularly in populations that are more sensitive to radiation exposure, such as children and young adults [18].

Future studies should explore the cost-effectiveness of DCE-CT compared to CE-CT, considering the potential reduction in additional diagnostic procedures and the overall impact on healthcare costs. Additionally, integrating advanced machine learning algorithms with DCE-CT data could further enhance lesion characterization and diagnostic accuracy, providing more precise and individualized patient care. While the slightly higher radiation dose warrants consideration, the substantial diagnostic benefits support the use of DCE-CT in clinical practice. Further prospective studies are essential to validate these findings and explore the broader benefits of DCE-CT in various clinical settings [19].

Overall, the results underscore DCE-CT's superior diagnostic efficacy compared to CE-CT, demonstrating higher sensitivity, specificity, and accuracy. The ability of DCE-CT to capture multiple phases of contrast enhancement provides more detailed and dynamic insights into lesion characteristics, leading to better clinical outcomes and more informed treatment planning. This improved diagnostic performance suggests that DCE-CT should be considered a valuable tool in the clinical evaluation of neck lesions.

## Conclusions

DCE-CT offers superior diagnostic efficacy compared to CE-CT for imaging neck lesions with enhanced sensitivity, specificity, and accuracy. DCE-CT's ability to capture multiple phases of contrast enhancement allows for detailed lesion characterization, providing crucial quantitative data on tissue perfusion and blood volume, which are vital for assessing tumor angiogenesis and therapy response. These features lead to more frequent improvements in patient outcomes and changes in treatment plans, demonstrating DCE-CT's significant clinical impact. The higher inter-observer agreement with DCE-CT also indicates better consistency among radiologists, which is crucial for reliable diagnosis and treatment planning.
